# Integrated Metagenomic and Metabolomic Profiling Identifies Severity-Specific Gut Microbiota Signatures Across A-B-E Phenotypes in Clinically Stable COPD: A Cross-Sectional Study

**DOI:** 10.3390/microorganisms14081673

**Published:** 2026-07-30

**Authors:** Renyang Tong, Li An, Ziting Liang, Yanan Wang, Xiaoshuang Lyu, Hengmo Rong, Yu An, Ruiyue Gao, Xiaoyan Liu, Zhaohui Tong, Chao Ren

**Affiliations:** 1 Department of Respiratory and Critical Care Medicine, Beijing Institute of Respiratory Medicine, Beijing Chao-Yang Hospital, Capital Medical University, Beijing 100020, China; tongry0329@hotmail.com (R.T.); bjzy818@sina.com (L.A.); lzt0615@126.com (Z.L.); wyn960202@sina.com (Y.W.); lvxiaoshuang2016@163.com (X.L.); ronghengmo@sina.com (H.R.); ruiyuegcherry@gmail.com (R.G.); lxycyyy@mail.ccmu.edu.cn (X.L.); 2 Beijing Key Laboratory of Multimodal Intelligent Diagnosis and Treatment System for Respiratory Diseases, Beijing Chao-Yang Hospital, Capital Medical University, Beijing 100020, China; 3 Medical Research Center, Beijing Institute of Respiratory Medicine, Beijing Chao-Yang Hospital, Capital Medical University, Beijing 100020, China; anyu900222@126.com; 4 Laboratory for Clinical Medicine, Capital Medical University, Beijing 100020, China; 5 Beijing Research Center for Respiratory Infectious Diseases, Beijing 100020, China

**Keywords:** chronic obstructive pulmonary disease, ABE groups, gut microbiota, metabolites

## Abstract

The ABE classification is crucial for the management of stable chronic obstructive pulmonary disease (COPD), reflecting disease symptom burden and exacerbation risk. Although gut microbiota is intimately linked to COPD pathogenesis, associations among the gut microbiota, its derived metabolites, and exacerbation risk in stable COPD patients remain poorly understood. We recruited 74 stable COPD patients (Group A, *n* = 18; Group B, *n* = 26; Group E, *n* = 30) for cross-sectional multi-omics profiling via fecal metagenomic sequencing and untargeted serum metabolomic analyses. Group E exhibited a decreasing trend in alpha diversity compared to Groups A and B. In addition, Group A displayed the most complex bacterial cooperative network, showing lower complexity and connectivity as symptom burden and risk of exacerbations increased. Taxonomically, the family *Prevotellaceae* was significantly enriched in Group A, while *Streptococcaceae* and *Lactobacillaceae* were more abundant in Groups B and E. Among 51 species displaying progressive trends with increasing exacerbation risk, 35 increased (e.g., *Clostridium ljungdahlii*) and 16 decreased (e.g., *Prevotella dentalis*). Furthermore, metabolomics analysis revealed that serum O-phosphoethanolamine levels were markedly elevated in Group E and showed a positive correlation with the COPD Assessment Test and modified Medical Research Council dyspnea scale scores. Exploratory mediation analysis suggested that elevated systemic O-phosphoethanolamine levels partially mediated the association between *Clostridium ljungdahlii* and COPD exacerbation risk. This study establishes significant associations between gut microbiota and phenotypic stratification in stable COPD patients. The identified *Clostridium ljungdahlii*/O-phosphoethanolamine axis may be associated with symptom burden and COPD exacerbation risk, provide a basis for further mechanistic studies.

## 1. Introduction

Chronic obstructive pulmonary disease (COPD) is characterized by irreversible bronchial and alveolar damage that manifests as persistent airflow limitation, frequent acute exacerbations, and progressive impairment of pulmonary function [1]. Widely recognized as a major global health threat, COPD affects over 400 million individuals and continues to be the third leading cause of global mortality [2]. The classification of COPD patients has been revised multiple times to improve clinical management [3]. Currently, the ABE classification system is recommended by the newest Global Initiative for Chronic Obstructive Lung Disease (GOLD) guidelines and considered an essential tool for the clinical stratification of COPD patients [3]. This classification relies on patient exacerbation history and respiratory symptom severity, as evaluated by the modified Medical Research Council (mMRC) dyspnea scale or the COPD Assessment Test (CAT). Furthermore, the ABE assessment tool is essential in the initial standardized management of COPD patients, especially for the selection of pharmacological treatments [3]. Therefore, clarifying risk factors for the development of ABE groups holds significance in deepening our understanding of COPD, and may assist in identifying potential therapeutic targets.

The gut microbiota constitutes the largest human microbial library, comprising over 100 trillion bacteria, as well as three million unique functional genes [4]. Previous research has identified the critical role of the gut microbiota in the pathogenesis of various diseases, such as neurodegenerative diseases, cardiovascular diseases, diabetes mellitus, and malignant tumors [5,6,7,8,9]. Notably, gastrointestinal disorders are intimately linked to both the pathogenesis and prognosis of COPD. For example, COPD patients showed a higher incidence of inflammatory bowel disease (IBD), encompassing both Crohn’s disease and ulcerative colitis [10]. In addition, IBD independently increases the risk of mortality among patients with COPD [11]. The gut–lung axis holds significant potential for clarifying pathogenic mechanisms, identifying early predictive biomarkers, and discovering novel therapeutic targets. Extensive research has confirmed the impact of gut microbiota on COPD, covering disease susceptibility, pathogenesis, severity prediction, and treatment [12]. Nonetheless, the associations among the gut microbiota, its derived metabolites, and exacerbation risk in stable COPD patients remain poorly characterized. Accordingly, the present study was designed to clarify the role of the gut microbiome in stratifying COPD patients based on the ABE assessment tool by analyzing microbial taxa and their metabolites.

## 2. Methods

### 2.1. Study Design and Ethics

**Figure 1:**
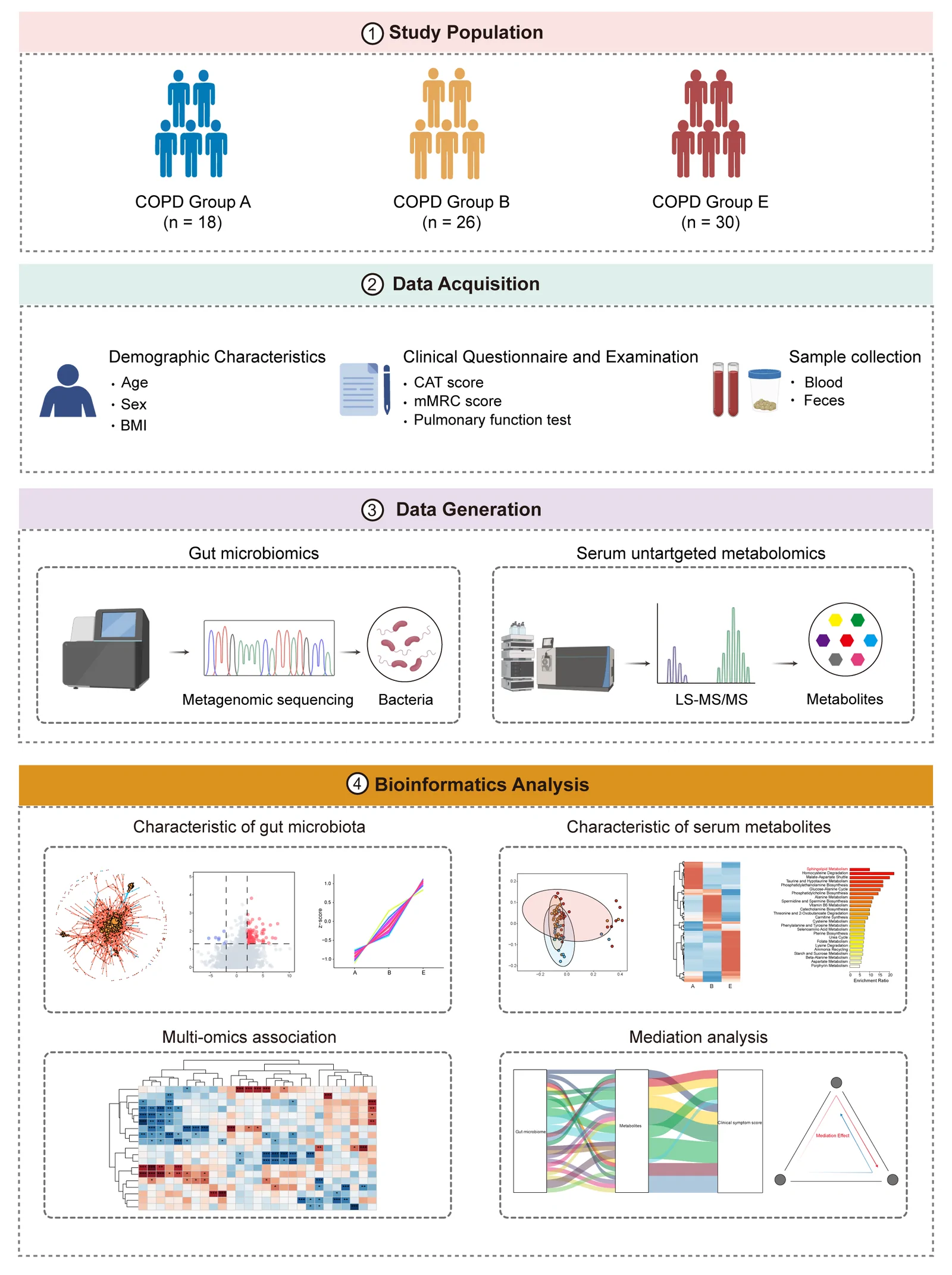
Schematic workflow diagram of this study. A graphical representation summarizing the study population, data acquisition, data generation, and bioinformatics analysis performed in 148 samples, including 74 fecal and 74 serum samples. COPD, chronic obstructive pulmonary disease; BMI, body mass index; CAT, COPD Assessment Test; mMRC, modified Medical Research Council. (* *p* < 0.05, ** *p* < 0.01, *** *p* < 0.001)

This research was conducted as a sub-cohort analysis within the Cohort Study for COPD in China (COMFORT), an ongoing, multicenter, prospective observational initiative aimed at evaluating the clinical characteristics of COPD (ClinicalTrials.gov Identifier: NCT03044847). To systematically investigate the gut microbiota and metabolic profiles of COPD patients with distinct A-B-E phenotypes, a standardized and reproducible workflow was developed (Figure 1). Participants with COPD were recruited from the Department of Respiratory and Critical Care Medicine, Beijing Chaoyang Hospital, between June 2023 and May 2024. Peripheral blood and fecal samples were collected from all participants. Ethical approval for this research was granted by the Ethics Committee of Beijing Chaoyang Hospital, Capital Medical University (Approval No: 2023-5-25-6 on 5 June 2023). All participants provided written informed consent before enrollment.

### 2.2. Study Cohort and Patient Characteristics

The criteria for inclusion were: (1) age above 40 years; (2) a confirmed COPD diagnosis based on the 2025 GOLD guidelines (post-bronchodilator FEV_1_/FVC < 70%); (3) no history of acute exacerbation in the prior 4 weeks. The exclusion criteria were: (1) concurrent asthma, current pulmonary tuberculosis, interstitial lung disease, or extensive bronchiectasis; (2) major comorbidities (e.g., active infection, diabetes mellitus, hyperlipidemia, cerebrovascular diseases, cardiovascular diseases, hepatic or renal dysfunction, cancer, or autoimmune disorders); (3) history of chronic intestinal disorder; (4) previous gastrointestinal surgical interventions; (5) exposure to probiotics or antibiotics within 4 weeks preceding sample collection; (6) administration of oral corticosteroids or traditional Chinese medicine within the previous 3 months; (7) being pregnant or lactating.

A total of 74 patients with COPD were recruited and further classified into Group A (*n* = 18), Group B (*n* = 26), and Group E (*n* = 30) based on the GOLD 2025 guidelines, which accounted for symptom burden and exacerbation risk. Symptom severity was assessed by using the mMRC (low = 0–1, high = ≥2) or CAT (low = <10, high = ≥10). Concurrently, exacerbation risk was classified into low (≤1 moderate exacerbation not requiring hospitalization) or high (≥2 moderate exacerbations or ≥1 severe exacerbation requiring hospitalization) categories. Based on this two-dimensional assessment, the cohort was delineated as follows: Group A included patients with low exacerbation risk and low symptom burden; Group B comprised patients with low exacerbation risk and high symptom burden; and Group E included patients with a high risk of exacerbation regardless of symptom burden level. All enrolled patients were in a stable phase of COPD. Detailed baseline characteristics were documented, including demographics, environmental air pollution exposure, COPD duration, smoking history, pharmacological treatments, laboratory tests, and dietary habits. Additionally, blood samples were collected and pulmonary function was assessed.

### 2.3. Fecal Sample Collection and Genomic DNA Extraction

Fresh fecal samples were collected from each participant, immediately transported to the laboratory on ice, and stored at −80 °C until processing. Genomic DNA was extracted via the cetyltrimethylammonium bromide (CTAB) method. The concentration of DNA was determined using the Qubit dsDNA HS Assay Kit on a Qubit 3.0 fluorometer (Invitrogen, Carlsbad, CA, USA). The integrity of DNA was subsequently assessed using 1% agarose gel electrophoresis.

### 2.4. The Collection, Preparation, and Storage of Serum

All peripheral venous blood samples were clotted at room temperature for 30 min, followed by centrifugation at 3000× *g* for 10 min. The resulting serum was aliquoted and preserved at −80 °C for subsequent experiments.

### 2.5. Metagenomic Sample Processing and Sequencing

Total fecal DNA was extracted using the Stool DNA Isolation Kit (Cat. No. DP712-02, TIANGEN). Sequencing libraries were generated using the TruSeq Nano DNA LT Library Preparation Kit (Cat. No. FC-121-4001, Illumina, San Diego, CA, USA). Genomic DNA was sheared via sonication to a target fragment size of 350 bp. The sheared DNA subsequently underwent end-repair, A-tailing, and adapter ligation. The adapter-ligated products were PCR-amplified under the following thermocycling conditions: an initial thermal denaturation at 95 °C for 3 min; followed by 8 amplification cycles with denaturation at 98 °C for 15 s, primer annealing at 60 °C for 15 s, and extension at 72 °C for 30 s; and a final extension step at 72 °C for 5 min. After library construction, fragment quality was verified using a Qsep-400 system. Subsequently, library concentration was quantified using a Qubit 3.0 fluorometer. The quality-controlled libraries were sequenced on an Illumina NovaSeq 6000 platform using paired-end 150 bp reads.

### 2.6. Metabolomic Data Acquisition and Processing

Metabolomic profiling of blood serum samples was performed using LC-QTOF/MS. Raw data underwent peak alignment, deconvolution, and integration with Agilent MassHunter Profinder (version B.10.0; Agilent, Santa Clara, CA, USA). Subsequent manual peak curation was conducted to eliminate background noise and validate integration accuracy for all retained molecular features. To correct systematic variation, data normalization was performed using a quality control (QC)-based systematic error removal using random forest (SERRF) algorithm. The preprocessed data were then filtered based on feature detection rate and reproducibility. Molecular features were retained if detected in at least 80% of samples within any study group and were excluded if the coefficient of variation (CV) > 20% in QC samples. The retained features were subsequently log-transformed and Pareto-scaled (Wilcoxon–Mann–Whitney test). Metabolites were identified via CEU Mass Mediator platform (http://ceumass.eps.uspceu.es, accessed on 14 April 2025) by matching their exact masses against the METLIN, KEGG, HMDB, and LipidMaps databases, accounting for isotopic patterns, adducts, and retention times. Further structural validation via MS/MS spectra was executed via MS-DIAL (version 4.8; https://systemsomicslab.github.io/compms/msdial/main.html, accessed on 14 April 2025), Lipid Annotator (version 10.0), and Agilent MassHunter. Manual verification was conducted when necessary, and identification confidence levels were determined in accordance with Metabolomics Standards Initiative (MSI) guidelines.

### 2.7. Metagenomic Analysis

Quality control of the metagenomic sequencing data was performed via FastQC (http://www.bioinformatics.babraham.ac.uk/projects/fastqc/, accessed on 15 August 2024). Raw sequencing reads were processed with Trimmomatic (v.0.39) to trim adapters and filter low-quality bases. Host-derived sequences were identified and depleted using Bowtie2 (version 2.4.4), yielding clean reads for subsequent analysis. The clean reads underwent de novo assembly to generate contigs via MEGAHIT (version 1.1.2), with contigs shorter than 500 bp discarded. Thereafter, genes were predicted from the qualified contigs using MetaGeneMark (version 3.26). CD-HIT was then applied to cluster sequences and reduce redundancy, using thresholds of ≥95% pairwise identity and ≥90% alignment coverage. Gene abundances across all samples were assessed using Bowtie2. Taxonomic profiling and functional annotation were performed by aligning the non-redundant genes with the NCBI non-redundant (nr) database and KEGG protein sequences using DIAMOND BLASTP (version 0.9.29.130). A taxonomic feature or a functional pathway was retained only if detected in ≥33% of samples, with a minimum total relative abundance of 0.01%.

Microbial taxonomic diversity was analyzed via α- and β-diversity assessments. α-diversity (Shannon, Chao1, ACE, and observed species richness indices) was calculated using the MicrobiotaProcess R package. Intergroup comparisons were performed using the Wilcoxon rank-sum test, with visualizations generated via ggplot2.

β-diversity was assessed by Bray–Curtis dissimilarity, and its patterns were visualized using principal coordinate analysis (PCoA). Subsequently, permutational multivariate analysis of variance (PERMANOVA) was conducted to evaluate variations in β-diversity among groups. Significant differential species were identified by the Wilcoxon–Mann–Whitney test and ANCOM-BC2 analysis with multiple-testing correction. In addition, microbial functional annotation was conducted using the KEGG Orthology (KO) database, and KEGG Level 3 pathway enrichment was analyzed via STAMP.

Microbial co-occurrence networks at the genus level were established based on Spearman’s rank correlations with the Hmisc package in R and displayed via Cytoscape (version 3.9.0). Only differentially abundant genera were retained for network construction. Edges in the network represent statistically significant pairwise correlations (*p* < 0.05 and |r| > 0.7), with orange and blue lines indicating positive and negative correlations, respectively. Node size was scaled proportionally to the number of significant interactions.

### 2.8. Metabolomic Analysis

PCoA was used to reduce data dimensionality and to evaluate metabolic differences among samples. *p*-values were derived by adjusting the Wilcox test via the Benjamini–Hochberg method. Metabolites with |log2 (fold change)| ≥ 1 and *p* < 0.05 were defined as differentially expressed metabolites (DEMs). Metabolic pathway enrichment analysis of DEMs was conducted using the KEGG database, and corresponding networks were constructed. Pathways were considered enriched only if mapped with more than three metabolites, and enrichment factors were calculated accordingly.

### 2.9. Association Analysis of Clinical, Metagenomic, and Metabolomic Data

Correlations among clinical respiratory scales, metagenomic data, and metabolomic data were evaluated. Specifically, clinical symptom burden was quantified utilizing the CAT and mMRC scores. Spearman’s rank correlation coefficients were computed through the Hmisc package and visualized as heatmaps with the pheatmap package in R.

### 2.10. Exploratory Bidirectional Mediation Analysis

To elucidate the mediation pathways linking gut microbial species, serum metabolites, and clinical symptom scores (CAT and mMRC), we first screened for candidate microbe-metabolite-symptom triads, requiring significant pairwise Spearman correlations among all three variables. Subsequently, exploratory bi-directional mediation analyses were conducted on these candidate triads using the R package “mediation” (version 4.5.0) to estimate the bidirectional mediation proportions and their statistical significance.

### 2.11. Statistical Analysis

All statistical analyses were performed using R software (version 4.4.2). Normally distributed continuous variables were expressed as mean ± standard deviation (SD), while non-normally distributed variables were presented as median with interquartile range (IQR). Intergroup comparisons of continuous variables were conducted using one-way analysis of variance (ANOVA) for normally distributed data, and the Kruskal–Wallis test for non-normally distributed data. Categorical variables were analyzed via the Chi-square test, and Fisher’s exact test was applied if expected frequencies < 5. Where applicable, age, gender, and body mass index (BMI) were adjusted as covariates. In this study, a *p*-value < 0.05 was considered statistically significant.

## 3. Results

### 3.1. Characteristics of the Participants

To comprehensively investigate the characteristics of gut microbiota in COPD patients with distinct A-B-E phenotypes, 74 COPD patients were enrolled following the inclusion and exclusion criteria and further divided into Group A (*n* = 18; low symptom burden/low risk), Group B (*n* = 26; high symptom burden/low risk) and Group E (*n* = 30; high exacerbation risk) (Figure 1). Seventy-four paired fecal and serum specimens from this cohort were subjected to shotgun metagenomic sequencing and untargeted metabolomic profiling, respectively (Figure 1). The baseline demographic and clinical features of the study participants are summarized in Table 1 and Table S1. The three phenotypic cohorts were well-matched, exhibiting no statistically significant variations in age, gender distribution, or BMI. Conversely, substantial intergroup disparities were observed regarding both symptom severity and pulmonary function parameters, including CAT and mMRC scores, as well as forced expiratory volume in 1 s (FEV1), forced vital capacity (FVC), and the FEV1/FVC ratio. During the clinically stable phase, 58 of 74 participants (78.4%) exhibited post-bronchodilator FEV1/FVC ratios <0.70, while 16 participants (21.6%) had ratios ≥ 0.70. Notably, patients in Group E exhibited the greatest symptom burden, as evidenced by the highest CAT and mMRC scores.

### 3.2. Rewiring of Microbial Interactions Among Gut Microbiota Across A-B-E Phenotypes in COPD Patients

**Figure 2:**
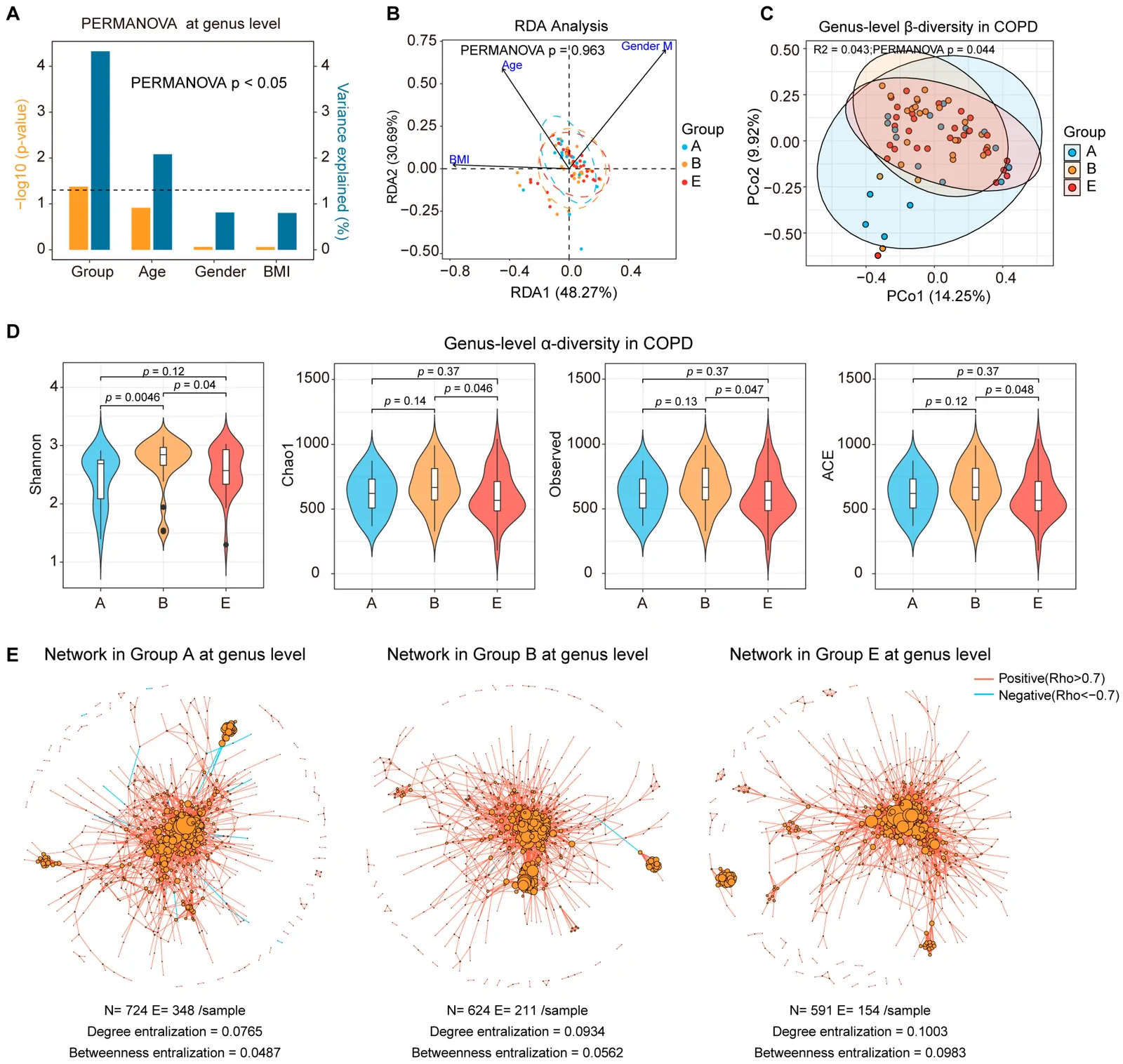
Gut microbial characteristics across A-B-E phenotypes in COPD patients. (**A**) Permutational multivariate analysis of variance (PERMANOVA) to account for confounding factors for gut microbial composition. (**B**) Redundancy analysis (RDA) analysis of gut microbial community structure and host factors (gender, age, and BMI). (**C**) Principal coordinate analysis (PCoA) of the Bray–Curtis distance at the genus level revealed the microbial compositional differences between Group A, Group B, and Group E. The statistical significance of the compositional differences among groups was evaluated via PERMANOVA. (**D**) Violin plots show the distribution of alpha diversity for each group at the genus level, with embedded boxplots representing the median and interquartile range. *p*-values were calculated via the Wilcoxon rank-sum test. (**E**) Microbial co-occurrence networks at the genus level for Group A (**left**), Group B (**middle**), and Group E (**right**). Pairwise correlations between microbial taxa were evaluated using Spearman’s rank correlation analysis. Only statistically significant correlations (|r| > 0.7, *p* < 0.05) were retained for network construction. Each node represents an individual microbial genus, with its size scaled proportionally to the number of significant inter-taxa correlations. Orange and blue edges denote positive and negative correlations, respectively. The number of nodes (N) and edges per sample (E/sample), degree centralization, and betweenness centralization are shown at the bottom of each panel. The size of each node represents its degree. Orange edges indicate positive correlations between microbial genera, whereas blue edges indicate negative correlations.

To comprehensively elucidate the relationship between the gut microbiome and COPD symptom burden and risk of exacerbations, high-throughput shotgun metagenomic sequencing was conducted across the cohort. Detailed information on gut microbiota sequencing data is displayed in Table S2. Species rarefaction curves were employed to assess and predict the extent to which species richness increases with sample size. The curve for three groups approached a plateau at higher sequencing depths, indicating adequate sampling and supporting the robustness of subsequent analyses (Figure S1). Thereafter, a PERMANOVA analysis was performed to account for major confounding factors. The results revealed that the disease state had significant effects on the composition of the gut microbiota at the genus level across A-B-E phenotypes (*p* < 0.05). These findings identified the COPD A-B-E phenotype as the key driver of microbial community structure in this COPD cohort (Figure 2A). Moreover, redundancy analysis (RDA) indicated that age, gender, and BMI collectively explained 30.69% of the variance in microbial composition; however, none of these covariates were significantly associated with community variation (PERMANOVA *p* = 0.963, Figure 2B). Moreover, the PCoA analysis using Bray–Curtis distances showed that the gut microbial structures varied significantly among the three groups (*p* = 0.044, Figure 2C). Additionally, Group E expressed lower alpha diversity compared with Group B (Figure 2D)**.** Co-occurrence network analysis at the genus level revealed progressive rewiring of microbial interactions across the A-B-E phenotypes in COPD (Figure 2E). Group A exhibited the most intricate network architecture (724 nodes and 348 edges per sample), reflecting a highly robust and interconnected ecosystem. Conversely, Group B displayed a sparser network with 624 nodes and 211 edges per sample, while Group E showed the least complex network (591 nodes, 154 edges per sample) with a modular structure and distinct taxon clusters. Meanwhile, both degree and betweenness centralization progressively increased from Group A to Group E. Specifically, betweenness centralization increased from 0.0487 to 0.0562 and 0.0983, while degree centralization increased from 0.0765 to 0.0934 and 0.1003. Collectively, these topological shifts signify a sequential collapse in microbial connectivity and ecosystem resilience that parallels the clinical advancement of COPD.

### 3.3. COPD Phenotype-Related Changes in Microbial Species Compositions

**Figure 3:**
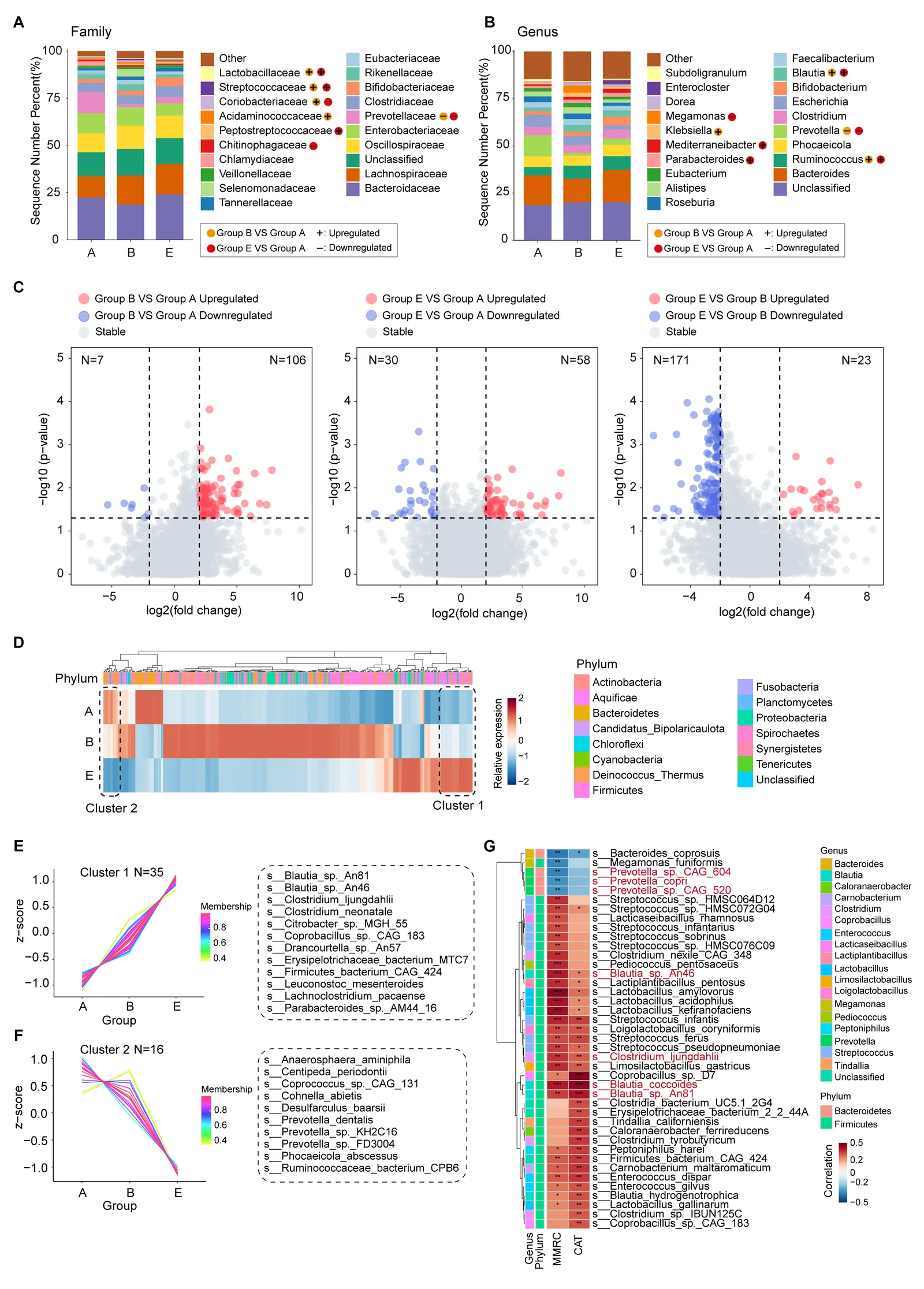
Alterations of the microbial species composition across A-B-E phenotypes in COPD. (**A**,**B**) Stacked barplots showing the distribution of the top 20 (**A**) families and (**B**) genera detected in the three groups. Each color represents a microbial taxon shown in the respective legend. Orange and red circles denote significant differences in Group B vs Group A and Group E vs Group A, respectively; “+” and “−” indicate increased and decreased relative abundance. (**C**) Volcano plots of the different species among the three groups (Group B vs. Group A, Group E vs. Group A, and Group E vs. Group B). Each dot corresponds to an individual microbial species. Blue dots represent downregulated species; red dots represent upregulated species; gray dots represent non-significant species. (**D**) Heatmap showing the relative abundance of differentially expressed species across the three groups. Red and blue indicate relatively higher and lower abundance, respectively. The colored annotation bar above the heatmap indicates the phylum classification of each species. The dashed boxes identify the species assigned to Clusters 1 and 2. (**E**,**F**) Mfuzz clustering analysis reveals two distinct trajectory clusters of gut microbial species across A-B-E phenotypes in COPD. Cluster 1: Species abundance positively correlated with A-B-E phenotypes in COPD; Cluster 2: Species abundance negatively correlated with A-B-E phenotypes in COPD. The dashed boxes list representative species from each cluster. (**G**) Heatmap showing Spearman’s rank correlations between gut microbiota and clinical parameters. Red and blue colors denote positive and negative correlations, respectively. The colored annotation bars indicate the genus and phylum classifications of the microbial species. Statistical significance is indicated within the squares (* *p* < 0.05, ** *p* < 0.01, *** *p* < 0.001).

To investigate the taxonomic shifts characterizing the distinct A-B-E phenotypes, the alterations of gut microbiota were analyzed from the family level down to the species level. Considerable differences in the gut microbial profiles were observed among the three groups. At the family level, *Prevotellaceae* was significantly enriched in Group A, and *Streptococcaceae* and *Lactobacillaceae* were more abundant in Groups B and E (Figure 3A and Table S3). At the genus level, the relative abundances of *Blautia* and *Ruminococcus* were elevated in Groups B and E, accompanied by a corresponding depletion of *Prevotella* (Figure 3B and Table S4). Considering that functional capacities can diverge substantially among species within a single genus, the subsequent analyses assessed compositional differences at the species level (Figure 3C, Tables S5 and S6). Hierarchical clustering of differentially abundant species delineated eight distinct abundance trajectories across the clinical groups (Figure 3D and Figure S2). Notably, the relative abundances of 35 species within Cluster 1 (e.g., *Blautia_*sp._An81, *Blautia_*sp._An46, *Clostridium_ljungdahlii,* and *Clostridium_neonatale*) increased with symptom burden and risk of exacerbations (Figure 3E and Table S5). In contrast, Cluster 2 (*n* = 16) comprised taxa such as *Prevotella dentalis*, *Prevotella* sp. KH2C16, and *Prevotella* sp. FD3004, which displayed a progressive decline as exacerbation risk and symptom burden increased (Figure 3F and Table S7).

To assess the clinical relevance of these microbial signatures, Spearman’s rank correlation analyses were performed to evaluate associations between specific taxa and pulmonary symptom scores (Figure 3G and Table S8). The correlation heatmap showed that five species were negatively correlated with CAT and/or mMRC scores, including three *Prevotella* species, one *Bacteroides* species, and one *Megamonas* species (Figure 3G). In contrast, 36 species showed positive correlations with symptom scores (Figure 3G). These positively correlated species were distributed across several genera, including eight *Streptococcus* species, four *Clostridium*-related species, four *Blautia* species, four *Lactobacillus* species and two *Enterococcus* species, suggesting that worsening respiratory symptoms were associated with broad and taxonomically diverse microbial alterations rather than with changes in a single genus. Collectively, these findings demonstrate associations between the intestinal microbiome and both symptom burden and exacerbation risk in COPD.

### 3.4. Alterations of the Microbial Functions Across A-B-E Phenotypes in COPD

**Figure 4:**
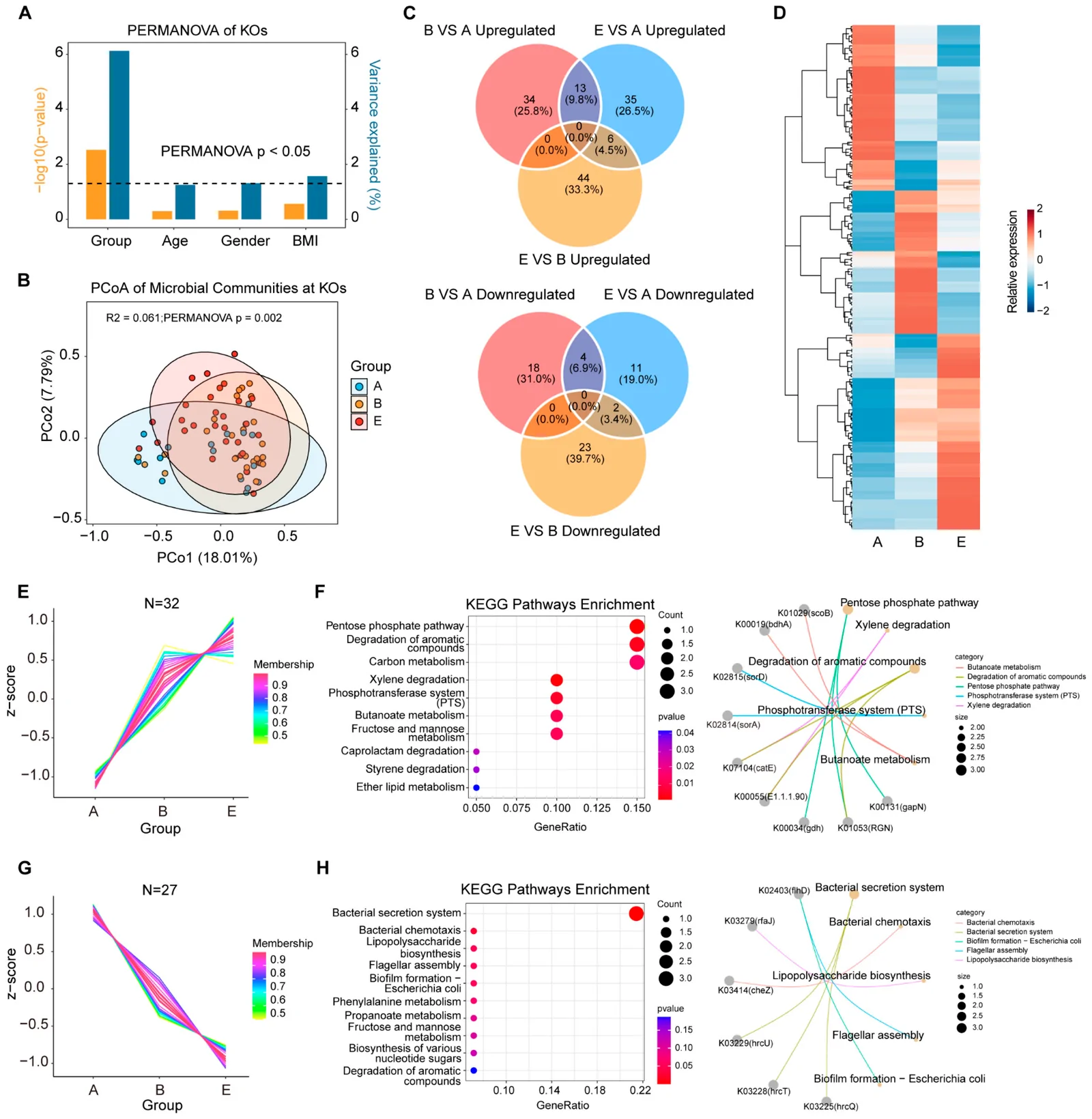
Alterations of the microbial functions across A-B-E phenotypes in COPD. (**A**) PERMANOVA was performed to assess the influence of potential confounding factors on microbial functional profiles. (**B**) PCoA highlighting the microbial functional differences at the KO level among the three groups. Statistical significance of group distributions was assessed using PERMANOVA. (**C**) Venn diagram of microbial functional features upregulated (upper) or downregulated (lower) in Group B vs. Group A, Group E vs. Group A, and Group E vs. Group B. (**D**) Heatmap of the relative abundance of differentially expressed KOs across the three groups. (**E**) Line plot showing the expression trajectories of KOs upregulated across A-B-E phenotypes in COPD. (**F**) Bubble diagram (left) for KEGG pathway enrichment analysis of KOs in (**E**). Gene ratio refers to the ratio of differential genes annotated to the total number of identified genes annotated in a given pathway. Network of associations between the top 5 KEGG pathways and related genes (right). (**G**) Line plot showing the expression trajectories of KOs downregulated across A-B-E phenotypes in COPD. (**H**) Bubble diagram (left) for KEGG pathway enrichment analysis of KOs in (**G**). Gene ratio refers to the ratio of differential genes annotated to the total number of identified genes annotated in a given pathway. Network of associations between the top 5 KEGG pathways and related genes (right). KEGG, Kyoto Encyclopedia of Genes and Genomes.

Following characterization of the taxonomic shifts, the functional capacities of the gut microbiome were evaluated across the three COPD groups. Metagenomic functional profiling successfully annotated a total of 4894 KEGG orthologs (KOs) in the cohort. Subsequent PERMANOVA analysis revealed that the clinical ABE classification was the only significant factor at the KO level (Figure 4A, *p* < 0.05). Mirroring the taxonomic compositional shifts, principal component analysis demonstrated significant spatial separation of microbial functional profiles among the groups (Adonis R = 0.061, *p* = 0.002, Figure 4B). To further delineate the functional alterations underlying these differences, gut microbial functional profiles were analyzed across A-B-E phenotypes in COPD patients (Figure 4C and Figure S3). Hierarchical clustering of the differentially abundant KOs was visualized via heatmap diagrams (Figure 4D and Table S9). Pattern analysis revealed that 32 KOs exhibited a gradual enrichment corresponding with increasing exacerbation risk (Figure 4E), whereas 27 KOs demonstrated a progressive depletion (Figure 4G). Functional enrichment analysis showed that KOs upregulated with increasing COPD exacerbation risk were primarily involved in the pentose phosphate pathway, phosphotransferase system (PTS), and butanoate metabolism (Figure 4F). Conversely, the KOs that decreased in abundance with increasing COPD exacerbation risk were primarily enriched in pathways related to the bacterial secretion system and bacterial chemotaxis (Figure 4H). Collectively, these functional perturbations indicate an association between gut microbial metabolic dysregulation and exacerbation risk of COPD.

### 3.5. Alterations of Serum Metabolome Across A-B-E Phenotypes in COPD Patients

**Figure 5:**
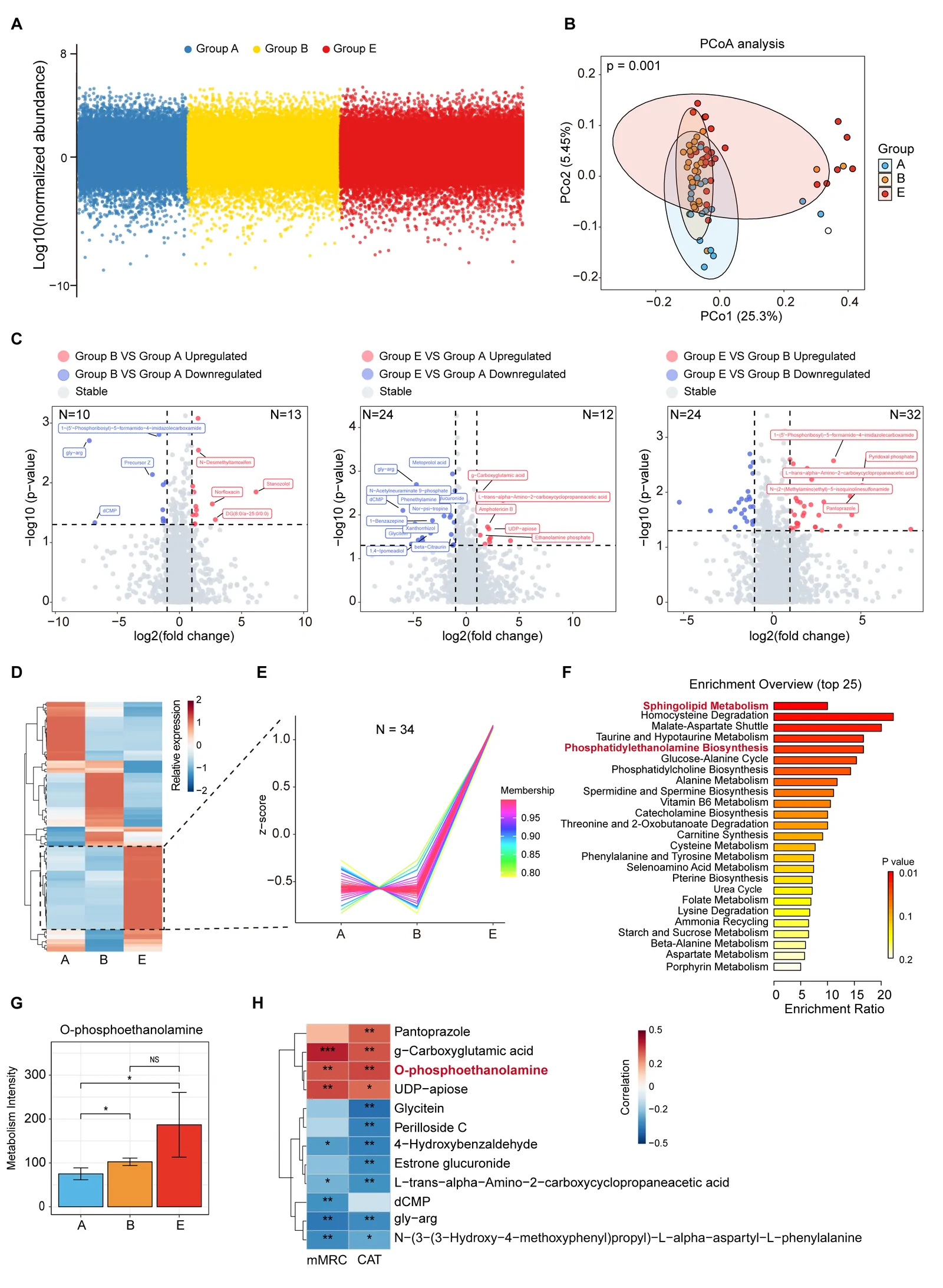
Alteration of serum metabolome across A-B-E phenotypes in COPD. (**A**) Boxplots illustrating the median and interquartile range of normalized metabolites abundance (*n* = 74). (**B**) PCoA of serum metabolites highlighting the overall metabolic differences between the three groups. Statistical significance of group separation was assessed using PERMANOVA. (**C**) Volcano plots of the different serum metabolites among the three groups (Group B vs. Group A, Group E vs. Group A, and Group E vs. Group B). Each dot corresponds to an individual serum metabolite. Red and blue dots denote significantly increased and decreased metabolites, respectively, while gray dots indicate non-significant changes. (**D**) Heatmap showing the relative abundance of the differentially expressed metabolites (DEMs) across the three groups. The dashed box indicates metabolites upregulated across A-B-E phenotypes in COPD. (**E**) Line plot showing the expression trajectories of DEMs upregulated across A-B-E phenotypes in COPD. (**F**) SMPDB functional enrichment analysis results of differential metabolites in (**E**). (**G**) Bar plot showing the expression levels of the O-phosphoethanolamine in the three groups. (**H**) Heatmap showing Spearman’s rank correlations between DEMs and clinical parameters. Red and blue colors denote positive and negative correlations, respectively. Statistical significance is indicated within the squares (* *p* < 0.05, ** *p* < 0.01, *** *p* < 0.001). NS, not significant; SMPDB, Small Molecule Pathway Database.

To elucidate the systemic metabolic consequences of COPD characterized by a high risk of exacerbations and a high symptom burden and their potential cross-talk with the gut microbiome, the serum metabolome was profiled utilizing untargeted LC-MS/MS. The distribution of normalized metabolite abundances was consistent across the three groups, indicating minimal batch-effect interference (Figure 5A). The PCoA analysis delineated clear spatial separation among the groups, with Group A clustering in closer proximity to Group B than to the high-risk Group E (Figure 5B). Subsequently, differences in metabolite abundances were compared among the three groups (Figure 5C and Table S10). Heatmap diagrams showed clusters of DEMs among three groups (Figure 5D). Specifically, 34 DEMs exhibited a gradual elevation with advancing ABE clinical groups (Figure 5E), and seven DEMs displayed a corresponding decrease (Figure S4A and Table S11). Next, these DEMs were analyzed for functional enrichment and showed significant enrichment in “Sphingolipid Metabolism” and “Phosphatidylethanolamine Biosynthesis” (Figure 5F). Notably, the expression level of O-phosphoethanolamine, a critical intermediate in lipid metabolism, was markedly upregulated, and this was associated with a higher risk of exacerbations (Figure 5G). In contrast, the levels of xanthorrhizol, a naturally occurring antibacterial compound derived from *Curcuma xanthorrhiza*, progressively declined with increasing exacerbation risk (Figure S4B).

To establish the clinical relevance of these circulating metabolic signatures, Spearman’s rank correlation analyses were performed. Serum O-phosphoethanolamine levels demonstrated a positive correlation with both CAT and mMRC symptom scores (Figure 5H and Table S12), suggesting an association between this metabolite and greater COPD symptom burden.

### 3.6. Interactions Between Gut Microbiota and Functional Metabolites in Blood

**Figure 6:**
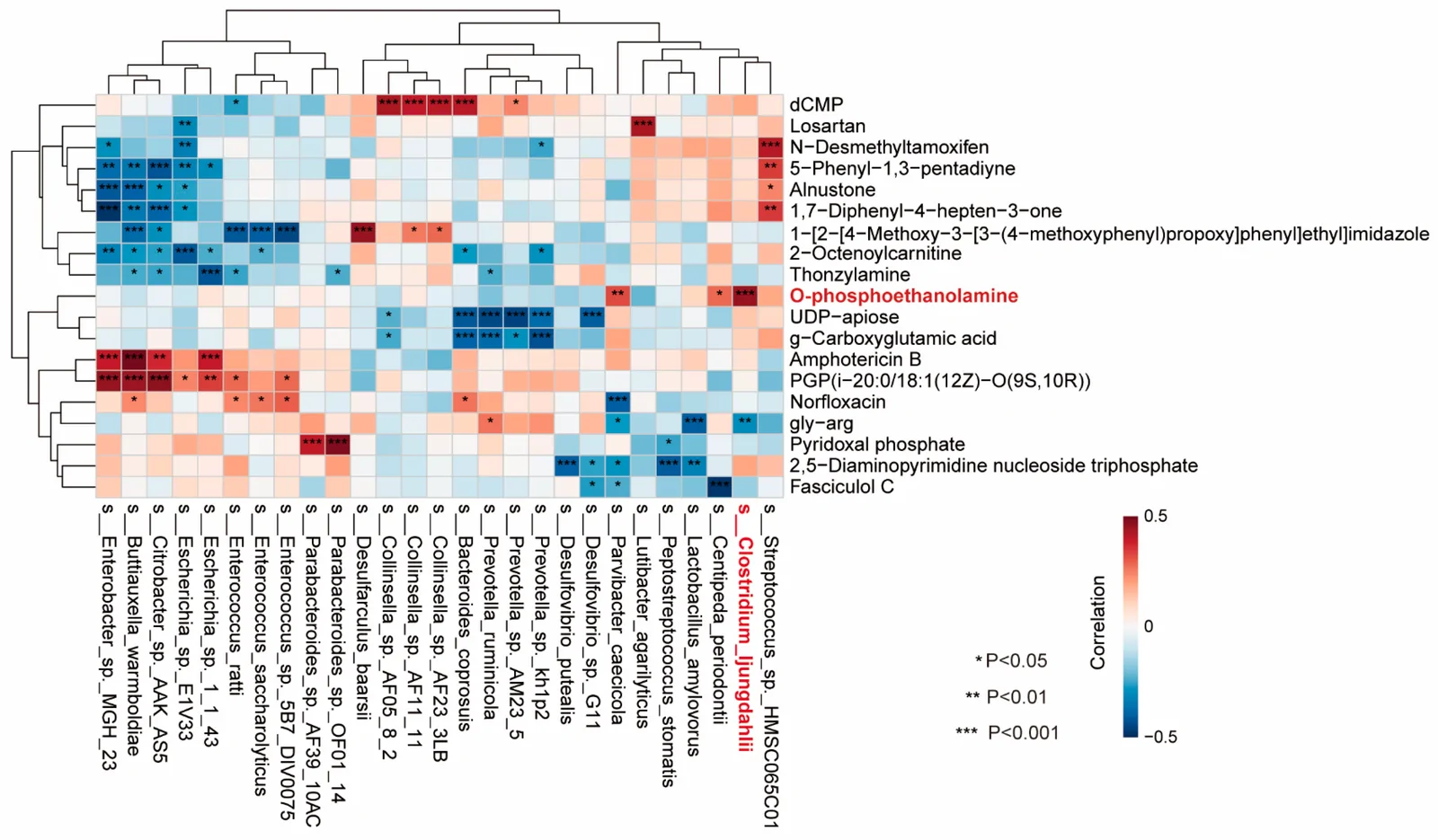
Heatmap showing the Spearman’s rank correlations between bacterial species and serum metabolites. Red and blue colors denote positive and negative correlations, respectively. Statistical significance is indicated within the squares (* *p* < 0.05, ** *p* < 0.01, *** *p* < 0.001).

To integrate the multi-omics datasets and map the intricate host–microbiome cross-talk, pairwise Spearman’s rank correlations were computed between the relative abundances of microbial taxa and serum metabolites. Thirty-two significant correlations (|r| > 0.4, *p* < 0.05) were identified between gut microbial taxa and serum metabolites (Figure 6 and Table S13). Specifically, circulating O-phosphoethanolamine exhibited a strong positive correlation with the abundance of *Clostridium ljungdahlii* (highlighted in red), a bacterial taxon previously documented for its ability to synthesize ethanolamine [13].

**Figure 7:**
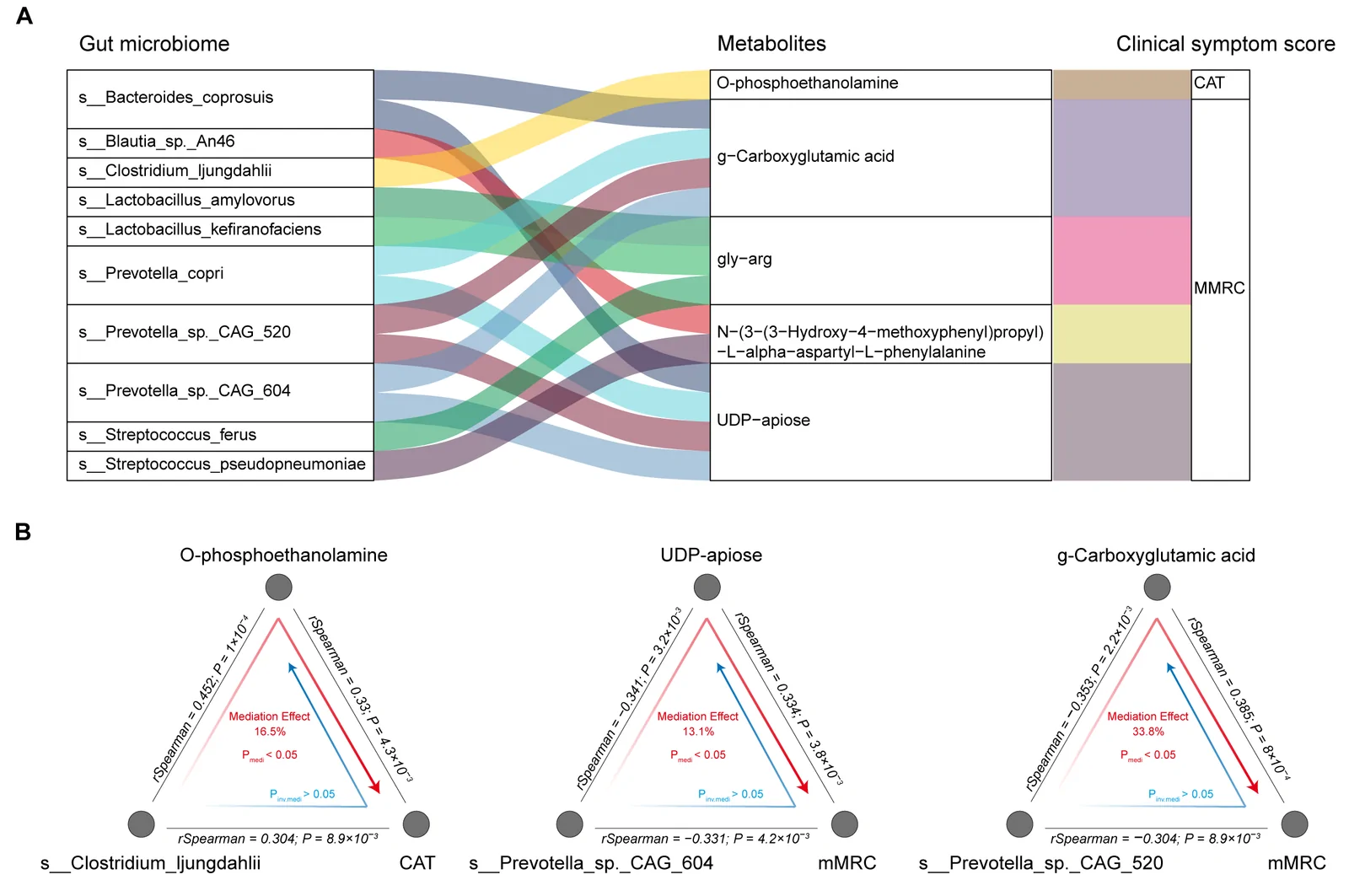
Mediation linkages among the gut microbiome, serum metabolites, and clinical features. (**A**) Sankey diagram depicting 14 significant mediation pathways linking the gut microbial features, serum metabolites, and clinical symptom scores. Columns represent microbial features (left), serum metabolites (middle), and clinical symptom scores (right). Connecting curved lines denote significant mediation effects, with different colors corresponding to distinct microbes and metabolites. (**B**) Representative mediation pathways linking gut microbial features, serum metabolites, and clinical symptom scores, as inferred via bi-directional mediation analysis. Spearman correlation coefficients and *p* values are annotated along each edge, with the proportions of mediation effects displayed at the center of the pathway diagrams. Red arrows indicate the effects of gut microbial features on clinical symptom scores mediated by serum metabolites, whereas blue arrows denote the reverse mediation (gut microbial effects on serum metabolites mediated by clinical symptom scores). The mediation significance (P_medi_ and P_inv.medi_) is derived from exploratory bi-directional mediation analysis.

To determine whether specific circulating metabolites potentially mediate the association between the gut microbiota and symptom burden in COPD, exploratory bi-directional mediation analyses were conducted. Exploratory mediation analysis uncovered 14 mediation linkages (P_mediation_ < 0.05, P_inverse mediation_ > 0.05, Figure 7A and Table S14). The mediation analysis revealed that O-phosphoethanolamine partially mediated the association between *Clostridium ljungdahlii* abundance and both greater symptom burden and a higher risk of exacerbations in COPD (16.5%, P_mediation_ < 0.05, Figure 7B). This partial mediation might link gut microbial metabolism to alterations in host lipid pathway alterations during COPD progression.

## 4. Discussion

Accumulating evidence indicates that dysregulation of gut microbiota is pivotal to the pathogenesis and clinical deterioration of various systemic conditions [14]. Specifically, the gut–lung axis highlights the significance of cross-talk between the intestinal microbiota and the lungs, showing great potential in maintaining structural and functional homeostasis of the airway [15]. Bacterial translocation and effects of microbiota-derived metabolites represent key mechanisms contributing to changes in coagulation, inflammatory, and immune responses [16]. Dysbiosis of gut microbiota is commonly seen in COPD patients and is involved in COPD exacerbation and poor outcomes [17]. The ABE assessment tool is currently utilized for the initial assessment of COPD patients, which also serves as a prerequisite for the use of nebulization drugs [3]. In this study, the β diversity showed significant differences among COPD patients with A, B, and E phenotypes, suggesting varied composition as COPD progresses.

Furthermore, the bacterial species in the A, B, and E groups were screened, revealing that the genus *Prevotella* was considerably decreased in the E group relative to the A and B groups, including *Prevotella_dentalis*, *Prevotella_*sp._KH2C16, and *Prevotella_*sp._FD3004. The *Prevotella* is an anaerobic, Gram-negative, non-spore-forming bacterium that is abundant in both the oral cavity and the gastrointestinal tract [18,19], with the type species being *Prevotella copri*. It exhibits protective effects on the mucosal barrier and reduces inflammatory responses by producing short-chain fatty acids (SCFAs), and is involved in the development of inflammatory diseases, including rheumatoid arthritis (RA), Parkinson’s disease and diabetes [20,21]. However, the role of *Prevotella* remains controversial. Previous studies have reported enrichment in the pathogenesis and progression of various diseases, such as RA, cardiovascular diseases, liver diseases, and even cancer [22,23,24,25]. Currently, no study has directly investigated the relationship between specific *Prevotella* species, including *Prevotella_dentalis*, *Prevotella_*sp._KH2C16, and *Prevotella_*sp._FD3004 and COPD development. Our findings demonstrate that a reduced relative abundance of *Prevotella* correlates with greater symptom burden and a higher risk of COPD exacerbation. In contrast, a previous study by Yan et al. showed that increased levels of *Prevotella* were associated with acute COPD exacerbation [26]. However, that study did not clarify the specific subtype of *Prevotella* and involved relatively small sample sizes. Therefore, the specific role of *Prevotella* in clinically stable COPD patients requires further validation in longitudinal and mechanistic studies. Notably, different subtypes of *Prevotella* may exhibit disparate effects in inflammatory diseases. The colonization of *Prevotella intestinalis* was found to exacerbate intestinal inflammation by reducing short-chain fatty acids [27]. Conversely, *Prevotella histicola* alleviates DSS-induced colitis by reducing the production of pro-inflammatory cytokines and preserving the epithelial barrier [28]. Given its diverse functions, the effects of *Prevotella* should be confirmed with specific sub-types.

Additionally, the genera *Blautia* and *Clostridium* showed increased clustering in E group compared to A and B groups. Conventionally, *Blautia* is characterized as a beneficial, SCFA-producing commensal known to alleviate various metabolic and inflammatory diseases [29,30,31]. However, few studies have investigated the role and mechanism of *Blautia* in the pathogenesis and progression of COPD. While Pei et al. [32] previously observed increased gut *Blautia* in COPD patients receiving erythromycin, our species-level analysis provides a more granular perspective. Specifically, the relative abundances of *Blautia* sp. An81 and *Blautia* sp. An46 increased with advancing clinical ABE groups and were positively correlated with symptom burden. The increased abundance of these taxa may reflect either a compensatory response to inflammation or an expansion associated with the COPD-related intestinal environment. Given the strain-specific functions of gut microorganisms, further in vivo studies are needed to determine whether these taxa exert protective or pathogenic effects in COPD. The genus *Clostridium* is usually Gram-positive bacteria with more than 150 species, and most species grow only in the complete absence of oxygen [29]. The clinical relevance of *Clostridium* bacteremia varies across reported studies [30]. For example, *Clostridium subterminale* could cause septicemia in patients in an immunosuppressive state or with a malignant tumor [31,32]. In addition, *Clostridium spiroforme* is associated with spontaneous and antibiotic-associated enteric disease [33]. In the present study, increased levels of *Clostridium ljungdahlii* and *Clostridium neonatale* were observed in gut microbiota across the A, B, and E groups, suggesting an association with increased COPD exacerbation risk. However, no study has investigated the effects of *Clostridium ljungdahlii* and *Clostridium neonatale* in the progression of COPD. Hosny M et al. [34] reported that *Clostridium neonatale* was significantly associated with necrotizing enterocolitis. *Clostridium ljungdahlii* serves as a synthesis gas-fermenting bacterium and shows tolerance to oxygen when cultured in rich mixotrophic medium [35]. Given its metabolic characteristics, *Clostridium ljungdahlii* may be associated with phenotypic stratification in stable COPD patients by participating in the metabolism of phosphoethanolamine. This deduction was further supported by metabolite analysis, which showed significantly increased levels of O-phosphoethanolamine. The existing literature indicates that O-phosphoethanolamine modulates immune-inflammatory responses by modifying the lipid A and core oligosaccharide structures of bacterial lipopolysaccharide (LPS). These modifications facilitate pathogen evasion of host innate immune defenses (such as resistance to antimicrobial peptides) and regulate the Toll-like receptor 4 (TLR4)/MD2 signaling pathway [36]. Concurrently, its systemic accumulation has been associated with the suppression of CD8+ T-cell function, potentially compromising host immune surveillance [37]. In the present study, circulating O-phosphoethanolamine levels were positively associated with CAT and mMRC scores, suggesting a relationship with COPD symptom burden. However, these findings should not be interpreted as direct evidence of causality. Experimental validation is required to determine whether O-phosphoethanolamine is functionally involved in the pathophysiology of COPD or primarily reflects disease-associated metabolic alterations. Although circulating O-phosphoethanolamine significantly mediated the association between *C. ljungdahlii* and COPD exacerbation risk, the modest proportion of mediation suggests that it is only one component of the broader gut–lung axis. Given the heterogeneous and multifactorial nature of COPD [38], this pathway may nevertheless represent a specific and biologically relevant metabolic link. The remaining unexplained association may involve additional microbial metabolites, co-occurring microbial taxa, inflammatory signaling, epithelial barrier dysfunction, immune-cell activation, environmental exposures, and host metabolic responses [12,39,40]. Therefore, the *C. ljungdahlii*–O-phosphoethanolamine pathway should be regarded as a potentially relevant biological link rather than the sole or predominant causal pathway.

Nevertheless, the limitations of this research should be acknowledged. First, the absence of a healthy control group precludes the identification of COPD-specific signatures. Future studies should therefore include healthy individuals to validate our findings and further strengthen the generalizability of our conclusions. Second, the study included a small sample of COPD patients from a single center, which limits statistical power; therefore, a multi-center study with a larger sample size might yield more robust results. Third, the clinical correlations were based primarily on CAT scores; future research should incorporate comprehensive clinical outcomes, including dynamic pulmonary function, exacerbation frequencies, and mortality rates. The results of metabolite analysis in fecal samples and targeted metabolomics were less stable in our study, emphasizing the need for further validation. Without concurrent evaluation of systemic inflammatory markers and rigorous in vitro or in vivo functional validation, the proposed mechanisms remain statistical hypotheses. Finally, technical constraints include potential variations in fecal metabolite stability and the inability to obtain absolute quantification of specific metabolites, which will require future targeted metabolomic validation. In addition, due to institutional ethical policies regarding human genetic privacy, raw sequencing data cannot be publicly deposited.

## 5. Conclusions

In conclusion, the intestinal microbiome is closely linked to phenotypic differentiation in stable COPD. High-risk (Group E) patients exhibit diminished microbial diversity and a significant depletion of the *Prevotella* genus. Notably, the enrichment of *Clostridium ljungdahlii* is significantly associated with greater COPD symptom burden and exacerbation risk, with the effect partially mediated by circulating O-phosphoethanolamine. These results provide novel perspectives on the “gut–lung axis” and highlight the *Clostridium ljungdahlii*–O-phosphoethanolamine pathway as a potential biomarker and target for future COPD mechanistic studies.

## Figures and Tables

**Table 1:** Baseline characteristics of included participants.

Characteristics	Group A (*n* = 18)	Group B (*n* = 26)	Group E (*n* = 30)	*p*-Value
Demographic data				
Age (years)	68.33 ± 7.84	67.54 ± 7.75	70.33 ± 6.23	0.333
Gender male	15 (83.33%)	21 (80.77%)	25 (83.33%)	>0.999
BMI (kg/m^2^)	24.37 ± 3.44	24.32 ± 4.01	22.87 ± 2.85	0.203
Clinical questionnaires				
CAT score	8.24 ± 3.31	17.29 ± 5.11	21.41 ± 6.52	<0.001 ^a,b,c^
mMRC score	0.94 ± 0.24	2.50 ± 0.83	2.55 ± 0.87	<0.001 ^a,b^
Pulmonary function test				
FEV1 (L) *	1.61 ± 0.58	1.31 ± 0.43	1.00 ± 0.40	<0.001 ^b,c^
FEV1 (% predicted) *	56.92 ± 25.04	47.74 ± 18.82	49.56 ± 22.09	0.387
FVC (L) *	2.92 ± 0.73	2.64 ± 0.65	2.36 ± 0.63	0.028 ^b^
FVC (% predicted) *	80.92 ± 21.25	79.34 ± 17.34	81.96 ± 24.50	0.908
FEV1/FVC ratio *	64.72 ± 17.87	55.12 ± 14.57	53.28 ± 12.96	0.045 ^b^

BMI, body mass index; CAT, COPD Assessment Test; mMRC, modified Medical Research Council; forced expiratory volume in 1 s, FEV1; forced vital capacity, FVC. *p*-values were derived for all groups using one-way ANOVA for continuous variables and Fisher’s exact test for categorical variables. *p* ^a^ < 0.05 for COPD-A and COPD-B; *p* ^b^ < 0.05 for COPD-A and COPD-E; *p* ^c^ < 0.05 for COPD-B and COPD-E. *: post-bronchodilator. Pulmonary function data shown in Table 1 were measured at enrollment during the clinically stable state.

## Data Availability

The data that support the findings of this study are available from the corresponding author upon reasonable request.

## Supplementary Materials

The following supporting information can be downloaded at https://www.mdpi.com/article/10.3390/microorganisms14081673/s1: Figure S1: Species rarefaction curves of COPD subgroups; Figure S2: Additional trajectory clusters identified by Mfuzz clustering analysis; Figure S3: Volcano plots of the different KOs among the three groups (Group B vs. Group A, Group E vs. Group A, and Group E vs. Group B). Each dot corresponds to an individual KO. Red and blue points denote significantly upregulated and downregulated KOs, respectively, while gray points indicate non-significant differences; Figure S4: Expression trajectory and specific metabolite levels downregulated across A-B-E phenotypes in COPD. (A) Line plot showing the expression trajectories of DEMs downregulated across A-B-E phenotypes in COPD. (B) Bar plot showing the expression levels of the xanthorrhizol in the three groups; Table S1: Additional baseline characteristics of included participants; Table S2: Summary of sequencing data quality for each sample; Table S3: Family-level differential abundance analysis between groups; Table S4: Genus-level differential abundance analysis between groups; Table S5: Species-level differential abundance analysis between groups using Wilcoxon–Mann–Whitney test; Table S6: Species-level differential abundance analysis between groups using ANCOM-BC2; Table S7: Species composition of clusters associated with COPD exacerbations risk; Table S8: Correlation analysis of gut microbiota with clinical parameters; Table S9: Differential abundance of KOs between groups; Table S10: Differential metabolites analysis between groups; Table S11: Metabolite composition of clusters associated with COPD exacerbations risk; Table S12: Correlation analysis of differentially expressed metabolites with clinical parameters; Table S13: Correlation analysis of bacterial species with serum metabolites; Table S14: Significant mediation pathways identified by exploratory bidirectional mediation analyses between gut microbiota, circulating metabolites, and clinical features.

## Abbreviations

BMI	Body mass index
CAT	COPD assessment test
COPD	Chronic obstructive pulmonary disease
DEMs	Differentially expressed metabolites
FEV1	Forced expiratory volume in 1 s
FVC	Forced vital capacity
GOLD	Global Initiative for Chronic Obstructive Lung Disease
IBD	Inflammatory bowel disease
IQR	Interquartile range
KO	KEGG Orthology
LPS	Lipopolysaccharide
mMRC	Modified Medical Research Council dyspnea scale
PERMANOVA	Permutational multivariate analysis of variance
PTS	Phosphotransferase system
QC	Quality control
RA	Rheumatoid arthritis
RDA	Redundancy analysis
SCFAs	Short-chain fatty acids
SD	Standard deviation
TLR4	Toll-like receptor 4
