# Supplementary material for: Integrated Metagenomic and Metabolomic Profiling Identifies Severity-Specific Gut Microbiota Signatures Across A-B-E Phenotypes in Clinically Stable COPD: A Cross-Sectional Study
Source: Microorganisms. 2026 Jul 30;14(8):1673. doi: 10.3390/microorganisms14081673 (PMC13515379; doi:10.3390/microorganisms14081673)
Supplement: Supplementary file 1 [file microorganisms-14-01673-s001.zip › Supplementary Figure.pdf]

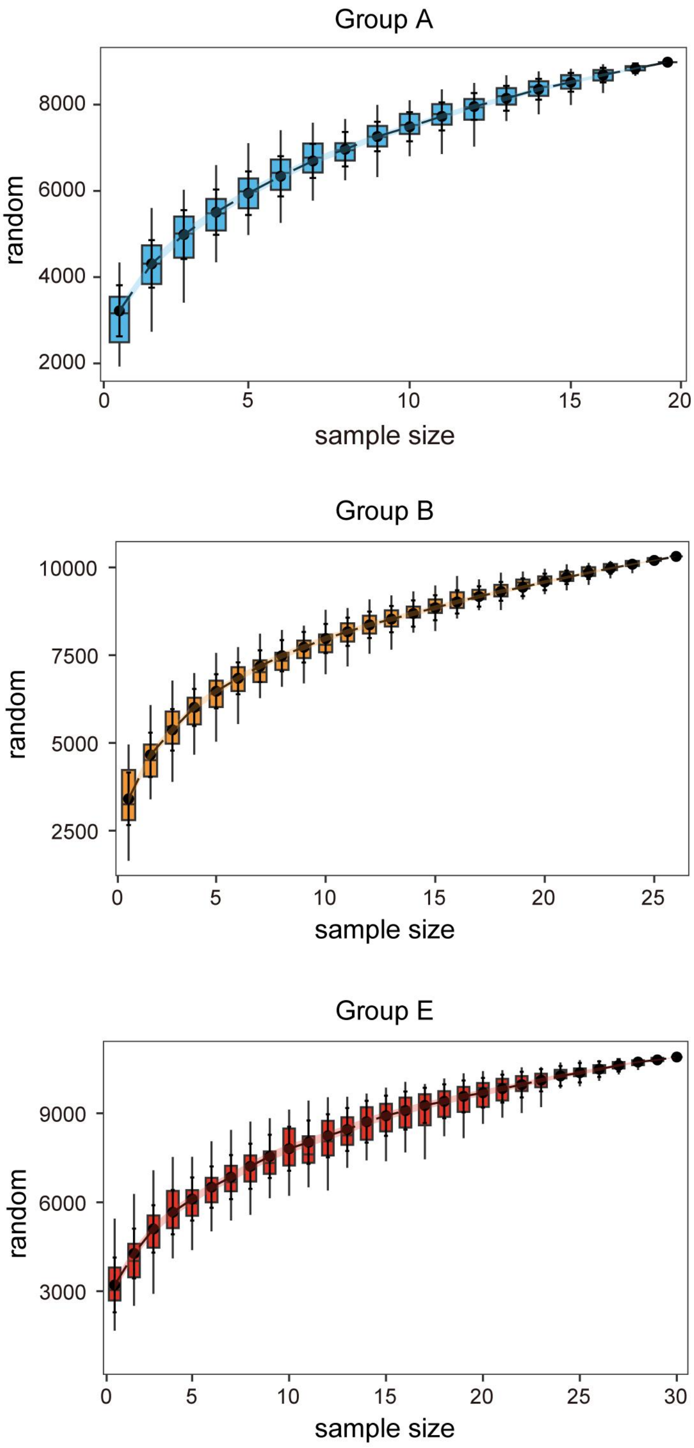

Figure S1. Species rarefaction curves of COPD subgroups.

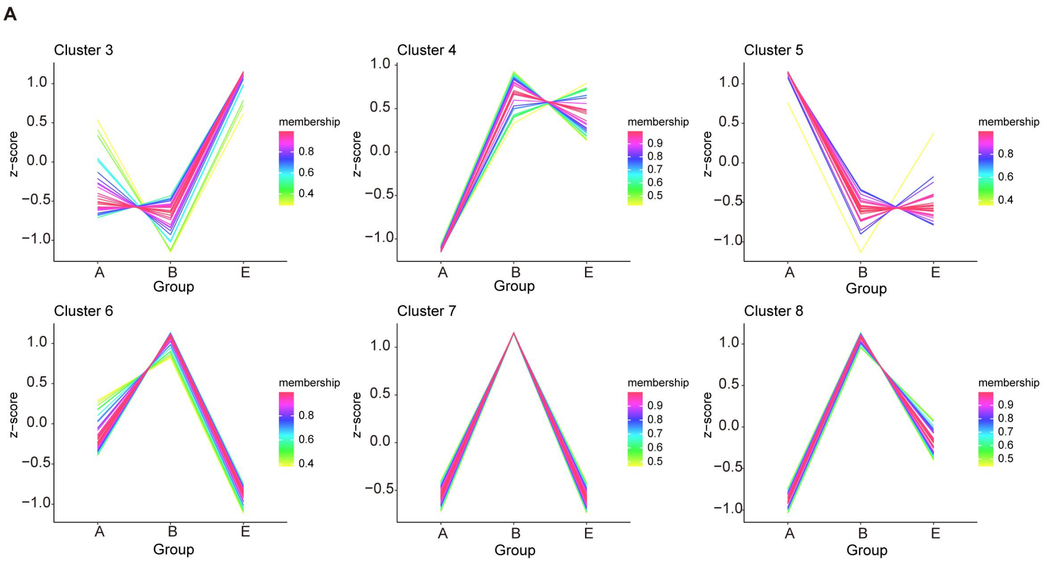

Figure S2. Additional trajectory clusters identified by Mfuzz clustering analysis.

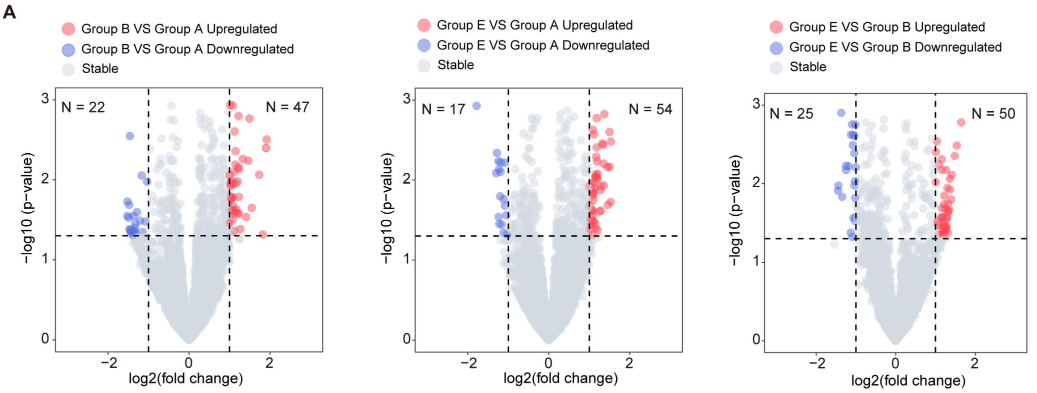

Figure S3. Volcano plots of the different KOs among the three groups (Group B vs. Group A, Group E vs. Group A, and Group E vs. Group B). The abscissa is the Fold change converted by log2, and the ordinate is the q-value converted by log10. Each dot represents a KO identified in the sample. Blue dots represent downregulated KOs; red dots represent upregulated KOs; gray dots represent non-significant KOs.

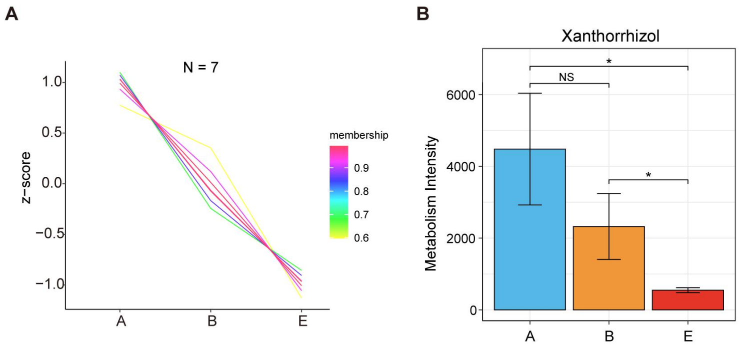

Figure S4. Expression trajectory and specific metabolite levels downregulated across A-B-E phenotypes in COPD.

(A) Line plot showing the expression trajectories of DEMs downregulated across A-B-E phenotypes in COPD.

(B) Bar plot showing the expression levels of the xanthorrhizol in the three groups.
