# Supplementary material for: Integrated Metagenomic and Metabolomic Profiling Identifies Severity-Specific Gut Microbiota Signatures Across A-B-E Phenotypes in Clinically Stable COPD: A Cross-Sectional Study
Source: Microorganisms. 2026 Jul 30;14(8):1673. doi: 10.3390/microorganisms14081673 (PMC13515379; doi:10.3390/microorganisms14081673)
Supplement: Supplementary file 1 [file microorganisms-14-01673-s001.zip › Supplementary Table S1.pdf]

Table S1: Additional baseline characteristics of included participants.

Characteristics	COPD-A (N = 18)	COPD-B (N = 26)	COPD-E (N = 30)	P-value
Smoking history, n (%)	14 (77.78%)	19 (73.08%)	24 (80.00%)	0.937
Pack-years of smoking	25.00 (19.20, 30.00)	16.00 (0.00, 30.00)	27.50 (0.00, 40.00)	0.502
Occupational exposure, n (%)	2 (11.11%)	3 (11.54%)	6 (20.00%)	0.704
Eosinophil ($\times 10^9$/L)	0.17 (0.08, 0.28)	0.15 (0.07, 0.22)	0.16 (0.07, 0.26)	0.704
Eosinophil (%)	2.90 (1.10, 3.70)	1.95 (1.30, 3.60)	2.15 (1.00, 4.30)	0.737
CRP (mg/dL)	0.41 (0.25, 0.50)	0.34 (0.23, 0.40)	0.34 (0.22, 0.45)	0.614
Treatment, n (%)				
LAMA	4 (22.22%)	3 (11.54%)	2 (6.67%)	0.283
LABA	2 (11.11%)	4 (15.38%)	0 (0.00%)	0.061
LABA+LAMA	3 (16.67%)	6 (23.08%)	4 (13.33%)	0.686
ICS+LABA	6 (33.33%)	5 (19.23%)	7 (23.33%)	0.511
ICS+LABA+LAMA	3 (16.67%)	8 (30.77%)	17 (56.67%)	0.014[b]
Exacerbation history, n (%)				
Exacerbation in the past year	5 (27.78%)	7 (26.92%)	30 (100.00%)	<0.001[b,c]
Severe Exacerbation in the past year	0 (0.00%)	0 (0.00%)	27 (90.00%)	<0.001[b,c]
Diet, n (%)				
Omnivore	16 (88.89%)	24 (92.31%)	26 (86.67%)	0.893
Vegetarian	1 (5.56%)	1 (3.85%)	3 (10.00%)	0.842
Vegan	0	0	0	>0.999
Pescatarian	0	0	0	>0.999
Other	1 (5.56%)	1 (3.85%)	1 (3.33%)	>0.999

CRP, C-reactive protein; ICS, inhaled corticosteroid; LABA, long-acting β2-agonist; LAMA, long-acting muscarinic antagonist.

Continuous variables were presented as means ± standard deviations (SD) if normally distributed or as medians with interquartile ranges (IQR) otherwise. Between-group differences for normally distributed continuous variables were assessed using one-way

analysis of variance (ANOVA), while the Kruskal-Wallis H-test was used for non-normally distributed variables. $\chi 2$ test were applied in comparing categorical variables and Fisher' s exact test were applied when the expected counts were less than 5. $P^a < 0.05$ for COPD-A and COPD-B; $P^b < 0.05$ for COPD-A and COPD-E; $P^c < 0.05$ for COPD-B and COPD-E.
